# Host-Adapted Ruminal Microbiota Investigation and Functional Validation of Duolang Sheep-Derived *Ligilactobacillus salivarius* KS1018

**DOI:** 10.3390/vetsci12121177

**Published:** 2025-12-10

**Authors:** Zixuan Wang, Yuchen Jia, Shiyu Duan, Hui Jiang, Cong Peng, Mingwei Mao, Yiping Zhu, Jing Li

**Affiliations:** 1 College of Veterinary Medicine, China Agricultural University, Beijing 100193, China; zixuanw@cau.edu.cn (Z.W.); jiayuchen@cau.edu.cn (Y.J.); jhcauvet@163.com (H.J.);; 2 State Key Laboratory of Veterinary Public Health and Safety, China Agricultural University, Beijing 100193, China; 3 Xinjiang Asman Animal Husbandry Co., Ltd., Kashgar 844100, China

**Keywords:** Duolang sheep derived *Lactobacillus salivarius* KS1018, mucosal immunity, rumen microbiota, 16S rRNA sequencing

## Abstract

This study characterized the rumen microbiota of Duolang sheep, a Xinjiang local breed, to identify potential probiotics for Hu sheep. 16S rRNA sequencing revealed that Duolang sheep possessed a distinct rumen microbial structure (beta-diversity, *p* < 0.05), lower diversity (higher Simpson index, *p* < 0.01), and a higher Firmicutes-to-Bacteroidetes ratio compared to Hu sheep. Subsequently, a Duolang-derived strain, *Ligilactobacillus salivarius* (*L. salivarius*) KS1018, was evaluated in a 56-day feeding trial with Hu sheep. Supplementation enhanced systemic immunity (serum IgG and IgM) and antioxidant status (SOD) (*p* < 0.05). Metabolic profiles were also modified, with serum β-hydroxybutyrate (BHB) level increasing significantly in both medium-dose (*p* < 0.001) and high-dose (*p* < 0.05) groups, whereas BUN and lipid markers (TG, CHOL) elevated significantly in the medium-dose (*p* < 0.05) and high-dose group (*p* < 0.05), respectively. The strain with medium- and high-dose also reduced fecal crude fat (*p* < 0.01) and influenced mucosal secretory immunoglobulin A (sIgA) by increasing levels in the duodenum while decreasing them in the rumen (*p* < 0.05). Ruminal histology was also altered (*p* < 0.01). Spearman’s correlation analysis showed that *Prevotellaceae_UCG-003* was positively correlated with serum BUN (*p* < 0.001), IgM (*p* < 0.05), BHB (*p* < 0.01), and SOD (*p* < 0.01). Overall, *L. salivarius* KS1018 improved antioxidant capacity, systemic and mucosal immunity, and ruminal structure in Hu sheep, demonstrating its potential to influence host–microbe interactions in livestock.

## Simple Summary

Duolang sheep, a native breed of Xinjiang, China, are well-adapted to local conditions, in contrast to the introduced, more fecund Hu sheep. This disparity poses a challenge for sustainable livestock production. We investigated whether the unique rumen microbiome of Duolang sheep could be a source of beneficial microbes to improve the health of Hu sheep. Our study first confirmed that the rumen microbiomes of the two breeds are distinct. We then tested the probiotic potential of a specific bacterium, *Ligilactobacillus salivarius* KS1018, isolated from Duolang sheep, as a feed supplement for Hu sheep. The supplement did not disrupt their overall gut microbe community, and it is beneficial to overall health, especially local gut immune function.

## 1. Introduction

The ability of ruminants to tolerate coarse feed and maintain health under challenging nutritional conditions is strongly related to their rumen microbiota [1,2]. This complex microbial ecosystem is essential for digesting feedstuffs, particularly the lignocellulosic carbohydrates that are otherwise inaccessible to the host animal [3,4]. The co-evolution between the host and its rumen microbiota plays a critical role in nutrient absorption and feed efficiency. The highly efficient rumen microbiota can lower the feed costs and increase profitability of sheep farms [5].

Duolang sheep, a local breed of in Xinjiang province, is a valuable genetic resource and exhibits remarkable ability to thrive with coarse feed [6,7,8]. However, their relatively low lambing rate (130%) limits their production efficiency. Consequently, the highly fecund Hu sheep, with lambing rates of 200–250%, have been introduced to Xinjiang [9,10]. However, it is challenging for the introduced Hu sheep to well adapt and efficiently utilize the low-quality local roughage, creating an urgent need for methods to enhance their digestive capabilities and resilience.

Their high feed efficiency indicates that Duolang sheep may possess a unique ruminal microbiota to facilitate roughage digestion. However, while whole-genome sequencing has been used to identify genetic markers for reproductive traits in this breed, the composition and function of its rumen microbiota remain largely unexplored [11]. Identifying and utilizing beneficial microbes is a promising strategy, as the gut microbiome critically influences the host’s immune status and growth performance [12,13].

Therefore, this study was designed to explore the microbial composition of Duolang sheep and compare the rumen microbiota to Hu sheep to identify unique ruminal microbial features. Based on the results, a potential probiotic strain, *Ligilactobacillus salivarius* KS1018 (*L. salivarius* KS1018), was isolated from Duolang sheep, and its effects on gastrointestinal and general health of Hu sheep were evaluated. This work is aiming to develop a type of functional probiotics, offering a sustainable alternative to antibiotics for improving the health of mutton sheep.

## 2. Materials and Methods

### 2.1. Ethics Statement

The experimental protocols were approved by the Animal Welfare and Ethics Committee of China Agricultural University (AW00213202-2-2). Written informed consent was obtained from the farm for animal participation.

### 2.2. Animals and Experimental Design

To characterize the breed-specific microbiota, rumen fluid was collected from 12 Duolang and 12 Hu ewes (18–24 months old) at a mutton sheep farm in Xinjiang. Subsequently, the feeding trial with Duolang-derived *L. salivarius* KS1018 was conducted with 32 male Hu sheep (105 days old, 30 ± 4 kg) at the same sheep farm. Prior to the experiment, all animals underwent health screening and were confirmed to be clinically healthy and free of gastrointestinal or systemic diseases. After a 14-day acclimatization to the facility and basal diet, the sheep were randomly assigned to four groups (*n* = 8). The experiment consisted of a 7-day pre-feeding period followed by a 56-day formal feeding trial. Treatments included a Control group (group C, receiving the basal diet without additive) and three *L. salivarius* KS1018 groups: Low (group L, 0.5 × 10^9^ CFU/sheep/day), Medium (group M, 1.0 × 10^9^ CFU/sheep/day), and High (group H, 1.5 × 10^9^ CFU/sheep/day). The probiotic powder (0.53 g/sheep/day, 1.05 g/sheep/day, and 1.58 g/sheep/day, respectively) was freshly mixed into the morning concentrate daily at 07:30. Dose levels were selected based on previous reports evaluating probiotic efficacy in ruminants [14]. All animals were group-housed identically with ad libitum access to water and a standardized total mixed ration (TMR; Supplementary Table S1).

### 2.3. Sample Collection

For the initial rumen microbiota comparison between breeds, rumen contents were collected via the left paralumbar fossa using aseptic techniques. After local disinfection and a small skin incision, a cannula was inserted into the ventral sac of the rumen. The samples were immediately stored on ice and sent to the lab for further processing. Approximately 50 mL of rumen contents were aspirated and immediately filtered through four layers of sterile cheesecloth and centrifuged at 8000 rpm for 10 min. The resulting liquid fraction (20 mL/sample) was preserved in liquid nitrogen for DNA analysis.

For the feeding trial, sample collection was conducted exclusively at the experimental endpoint (Day 56). Blood samples (10 mL) were collected from the jugular vein, and plasma was separated by centrifugation (3000× *g*, 4 °C, 15 min) and stored at −80 °C. Fecal samples (20 g) were collected from the rectum and stored in liquid nitrogen. Three Hu sheep of each group with body weights near the group average were selected, anesthetized, and euthanized for necropsy. Mucosal samples (200 mg each) were collected from the ventral sac of the rumen and middle portion of the ileum by scraping with a glass slide and subsequently transferred into 2 mL cryovials for further assessment.

### 2.4. L. salivarius KS1018 Preparation

The strain *L. salivarius* KS1018 was isolated from the rumen fluid of healthy Duolang sheep aged 18 months, sourced from Kashgar, Xinjiang. In vitro experiments related to probiotic traits including stress tolerance, antimicrobial activity and biochemical characteristics have been conducted [15]. For the feeding trial, *L. salivarius* KS1018 was prepared as a freeze-dried powder (≥1.0 × 10^9^ CFU/g) by Dingchuang Biotechnology (Dongying, China) using a multi-step, fed-batch fermentation process. The strain was activated and inoculated into a 100 L fermenter (60% working volume), with dissolved oxygen maintained at 30% via automated agitation (200–600 rpm) and aeration (1.0–1.2 vvm). The biomass was harvested via disc stack centrifugation, freeze-dried (−45 °C pre-freezing), and milled to a fine powder (80–100 mesh) to achieve a final concentration of 7.6 × 10^9^ CFU/g.

### 2.5. Serum Biochemical, Immunity and Hormonal Profiling

Serum biochemical parameters, including blood urea nitrogen (BUN), triglycerides (TG), and cholesterol (CHOL), were analyzed using an automated analyzer (VetScan HM5, Abaxis, Union City, CA, USA). Oxidative stress marker superoxide dismutase (SOD) was quantified using ELISA kits (Meimian, Yancheng, China). Additionally, β-hydroxybutyrate (BHB) level was measured with ELISA kits (Meimian, Yancheng, China). The serum concentrations of IgG and IgM were detected using enzyme-linked immunosorbent assay (ELISA) diagnostic kits (Huzhen Industrial Co., Ltd., Shanghai, China).

### 2.6. Fecal Characteristics Analysis

The physical characteristics of fresh feces from Hu sheep were observed daily, and fecal scoring was performed based on fecal morphology with reference to the Bristol Stool Form Scale (BSFS) [16]. For compositional analysis, fecal samples were collected and stored frozen at −80 °C. Samples of feces were dried at 65 °C in forced draft oven for about 72 h and ground to pass 1 mm mesh screen size. The ground samples were stored in airtight plastic containers pending chemical analysis. The moisture content and dry matter (DM) were determined according to the national standard GB 5009.3-2016 [17]. On a DM basis, the samples were subsequently analyzed for ash (GB/T 6438-2007) [18], crude protein (CP; GB/T 6432-2018) [19], ether extract (EE, for crude fat; GB/T 6433-2006) [20], crude fiber (CF; GB/T 6434-2006) [21], neutral detergent fiber (NDF; GB/T 20806-2006) [22], and acid detergent fiber (ADF; NY/T 1459-2022) [23]. Digestible energy (DE) was calculated according to the following formula: DE (kJ/100 g) = (100 − %Ash − %EE − %CP) × 17 + (%CP × 17) + (%EE × 37).

### 2.7. Histological Analyzes

Tissue samples were collected from the ventral sac of the rumen (2 cm × 2 cm squares) and the middle portion of the ileum (1 cm transverse segments). All samples were immediately fixed in 10% neutral buffered formalin, dehydrated in graded ethanol, and embedded in paraffin. Two paraffin sections (5 μm thickness) from each rumen and ileum sample were cut and stained with hematoxylin and eosin (H&E) staining kit (Boster Biological Technology, Wuhan, China). The stained sections were scanned using a digital slide scanner (KFBIO, Ningbo, China), and morphometric measurements were performed using K-viewer software (v1.7.1.1, KFBIO). Five non-overlapping and well-preserved microscopic fields for each tissue were randomly selected for quantitative measurement.

For rumen tissues, the measured parameters included stratum corneum thickness, non-keratinized epithelial thickness, connective tissue width, and total papilla width [24]. For ileal tissues, villus height (from the villus tip to the villus-crypt junction), crypt depth (from the crypt base to the villus base), and the villus-to-crypt ratio were recorded. For statistical analyses, the mean value of all ten microscopic fields per animal was used.

### 2.8. DNA Extraction and Sequencing

Genomic DNA was extracted from 24 samples using the E.Z.N.A.^®^ Soil DNA Kit (Omega Bio-tek, Norcross, GA, USA). DNA quality was verified via 1.0% agarose gel electrophoresis and NanoDrop2000 spectrophotometer (Thermo Scientific, Waltham, MA, USA). The V3-V4 hypervariable region of bacterial 16S rRNA was amplified using primers 338F (5′-ACTCCTACGGGAGGCAGCAG-3′)/806R (5′-GGACTACHVGGGTWTCTAAT-3′) on a GeneAmp PCR System 9700, version 3.12 (Thermo Scientific, Waltham, MA, USA). PCR conditions included: 95 °C for 3 min; 29 cycles of 95 °C (30 s), 53 °C (30 s), 72 °C (45 s); final extension at 72 °C (10 min). The PCR product was verified on 2% agarose gel and purified with the PCR Clean-Up Kit (YuHua, Shanghai, China). Then, purified amplicons were quantified by Qubit 4 Fluorometer (Thermo Fisher Scientific, Waltham, MA, USA) and sequenced on an Illumina PE300/PE250 platform (Illumina, San Diego, CA, USA).

### 2.9. Bioinformatic Processing

Raw FASTQ files were demultiplexed, quality-filtered (fastp v0.19.6), and merged (FLASH v1.2.11). Operational taxonomic units (OTU) were clustered at 97% similarity (UPARSE 11), with taxonomic classification performed using the Ribosomal Database Project (RDP) Classifier (v2.13) against the Silva v138 database (confidence threshold: 0.7). Functional predictions were generated via PICRUSt2. Phylogenetic placement used EPA-NG and Gappa, while 16S gene copies were normalized using Castor.

### 2.10. Statistical Analysis

Alpha diversity metrics (observed OTUs, Chao1, Shannon, Simpson, Good’ s coverage) and rarefaction curves were calculated in Mothur (v1.30.2). Beta diversity was assessed via principal coordinate analysis (PCoA) based on Bray–Curtis dissimilarities (Vegan v2.5-3). PERMANOVA tests evaluated group variations with 999 permutations. Differentially abundant taxa (phylum to genus) were identified using the linear discriminant analysis (LDA) effect size (LEfSe, LDA score > 2.5 or 4, *p* < 0.05). To control for false positives in multiple taxa comparisons, *p*-values were adjusted using the Benjamini–Hochberg false discovery rate (FDR) method. Spearman’s rank correlation analysis was performed to assess the associations between the relative abundance of top 20 genera and host serum parameters. The correlation heatmap was visualized using the Majorbio Cloud Platform. Other analyses were conducted using GraphPad Prism 10.3.0 (GraphPad Software, San Diego, CA, USA). All quantitative data are presented as means ± standard deviations (SD). The data were first assessed for normality using the Shapiro–Wilk test and homogeneity of variance. For normally distributed data with equal variances, one-way ANOVA was used, followed by Tukey’s multiple comparisons test to determine differences between groups. For non-normally distributed data, the Kruskal–Wallis test was applied, and Dunn’s multiple comparisons test was used for post hoc analysis. Statistical significance was set at *p* ≤ 0.05, and 0.05 < *p* < 0.10 was considered a statistical tendency.

## 3. Results

### 3.1. Comparison of Rumen Microbiota Between Hu Sheep and Duolang Sheep

A total of 1,675,979 sequence reads with an average length of 416 bp per sequence remained after primer removal, quality-filtering, and chimera-checking. A total of 12,684 OTUs were identified and assigned into 23 phyla, 53 classes, 112 orders, 181 families and 368 genera. 8797 and 8587 OTUs were detected in rumen fluid samples from Duolang sheep and Hu sheep, respectively.

**Figure 1:**
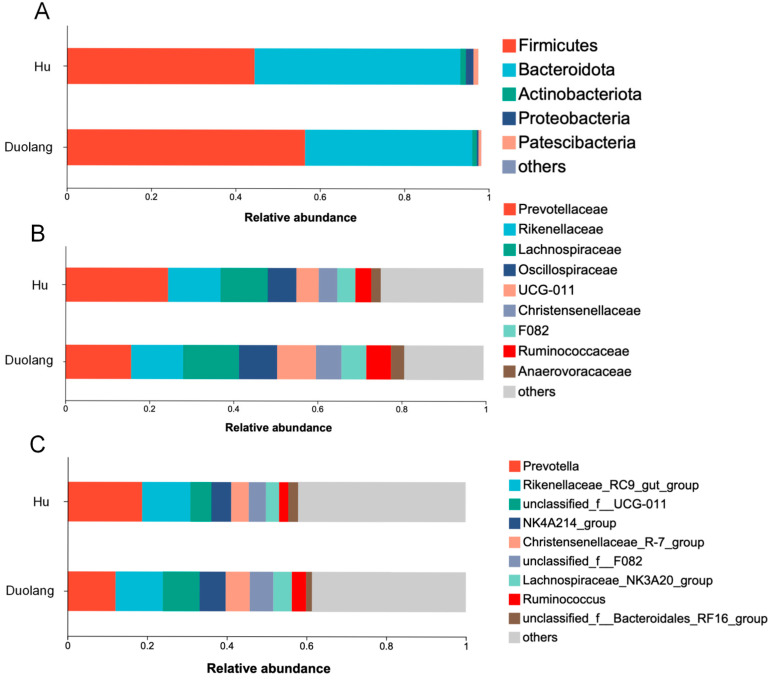
Relative abundance of rumen bacteria at the phylum (**A**), family (**B**), and genus (**C**) levels in Hu sheep (*n* = 12) and Duolang sheep (*n* = 12). Others: remaining bacterial taxa with lower relative abundance. Unclassified: sequences that could not be assigned to certain taxa.

There were 5 and 3 bacterial phyla, 19 and 16 bacterial families, 20 and 22 bacterial genera with relative abundance of >1% in ruminal fluid from Hu sheep and Duolang sheep, respectively. Significant differences and statistical tendencies in the relative abundance of dominant bacterial taxa were observed in the ruminal fluid between Hu and Duolang sheep after FDR correction (Table 1; Figure 1).

**Figure 2:**
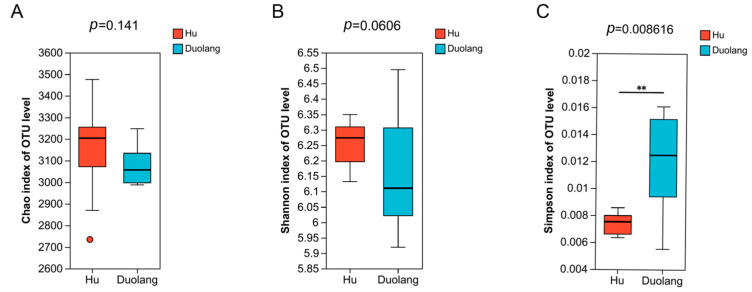
Alpha diversity indexes of ruminal microbiota of Hu sheep and Duolang sheep (*n* = 12). (**A**) Chao index of OTU level. (**B**) Shannon index of OTU level. (**C**) Simpson index of OTU level. ** *p* < 0.01.

There is no statistical difference in Chao richness and Shannon diversity between the two sheep breeds (Figure 2A,B; *p* > 0.05). The Simpson index of Duolang sheep is significantly higher than that of Hu sheep (Figure 2C; *p* = 0.0086).

**Figure 3:**
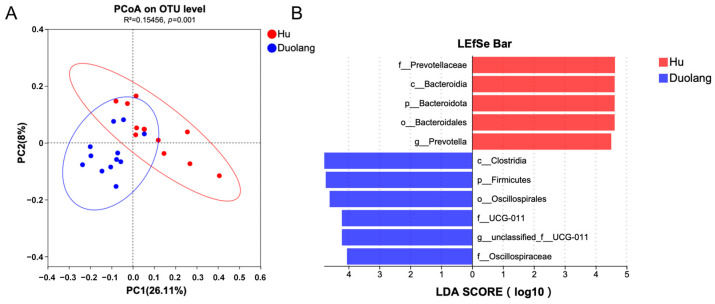
(**A**) PCoA of rumen microbiota between Hu and Duolang sheep based on Bray–Curtis distance. (**B**) Linear discriminant effect size analysis (LEfSe) of rumen microbiota between Hu and Duolang sheep demonstrating differentiating bacteria taxa from genus and higher (LDA score > 4, *p* < 0.05).

Beta diversity was calculated with Bray–Curtis dissimilarity and demonstrated by PCoA (Figure 3A). 482 out of the total 12,684 OTUs were significantly different between Hu and Duolang sheep (*p* < 0.05). Among the identified taxa from genus to phylum, 737 taxa were significantly different between Hu and Duolang sheep (*p* < 0.05). Among these taxa, 61 taxa had an LDA above 3, and 11 had an LDA score above 4 (Figure 3B).

### 3.2. Fecal Characteristics of Hu Sheep After L. salivarius KS1018 Supplementation

Supplementation with *L. salivarius* KS1018 resulted in significant differences in fecal crude fat content in Hu sheep (*p* = 0.0099) with Group M (4.43 ± 1.13%) showing significantly higher crude fat levels compared to Group H (2.63 ± 1.02%) and Group L (2.82 ± 1.29%) (Table 2). No significant differences were observed in other fecal components, fecal score, or digestible energy.

### 3.3. Serum Biochemical, Immunity and Antioxidant Profiles of Hu Sheep After L. salivarius KS1018 Supplementation

**Figure 4:**
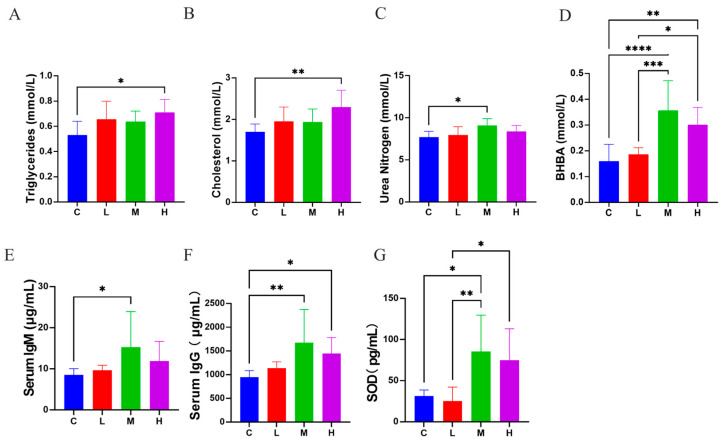
Effects of *L. salivarius* KS1018 supplementation on serum biochemical, immune, and antioxidant profiles in Hu sheep (*n* = 8). (**A**) Triglycerides (TG). (**B**) Cholesterol (CHOL). (**C**) Urea nitrogen (BUN). (**D**) β-hydroxybutyrate (BHB). (**E**) Serum IgM. (**F**) Serum IgG. (**G**) Superoxide dismutase (SOD). Data are presented as Mean ± SD. * *p* < 0.05, ** *p* < 0.01, *** *p* < 0.001, **** *p* < 0.0001.

Compared to group C, group H exhibited elevated TG (0.71 ± 0.10 vs. 0.53 ± 0.11 mmol/L; *p* < 0.05; Figure 4A) and CHOL concentrations (2.30 ± 0.41 vs. 1.70 ± 0.19 mmol/L; *p* < 0.01; Figure 4B). Nitrogen metabolism was affected, with group M showing significantly higher BUN concentrations compared to group C (9.06 ± 0.83 vs. 7.70 ± 0.70 mmol/L; *p* < 0.05; Figure 4C). Regarding ketone body metabolism, BHB concentrations were significantly elevated in group M (0.36 ± 0.12 vs. 0.16 ± 0.66 mmol/L; *p* < 0.0001; Figure 4D) and group H (0.30 ± 0.67 vs. 0.16 ± 0.66 mmol/L; *p* < 0.001; Figure 4D). IgM concentrations were significantly higher in group M (15.26 ± 4.78 vs. 8.53 ± 1.50 μg/mL; *p* < 0.05; Figure 4E). Similarly, IgG levels showed significant increases in both group M (1672.53 ± 701.52 vs. 948.50 ± 137.93) and group H (1447.34 ± 335.15 vs. 948.50 ± 137.93 μg/mL; *p* < 0.05 and *p* < 0.01; Figure 4F). SOD activity significantly increased in group M (85.36 ± 44.37 vs. 31.39 ± 7.25 pg/mL; *p* < 0.01; Figure 4G).

### 3.4. Mucosal sIgA Levels of Hu Sheep After L. salivarius KS1018 Supplementation

Supplementation with *L. salivarius* KS1018 exerted a segment-specific modulatory effect on mucosal secretory immunoglobulin A (sIgA) concentrations in the digestive tract of Hu sheep (Table 3). *L. salivarius* KS1018 supplementation significantly affected sIgA levels in the rumen and duodenum (*p* < 0.01), whereas no significant changes were observed in the jejunum and ileum (*p* > 0.05). Specifically, *L. salivarius* KS1018 supplementation significantly enhanced duodenal sIgA in group H (*p* < 0.01), while ruminal sIgA was significantly reduced in all *L. salivarius* KS1018 supplemented groups compared to group C (*p* < 0.05).

### 3.5. Histological Analyzes of Hu Sheep After L. salivarius KS1018 Supplementation

**Figure 5:**
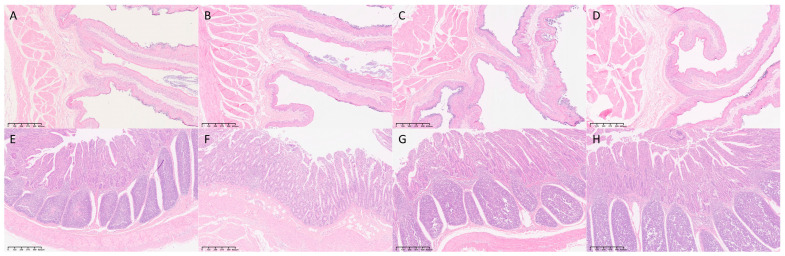
Histological analysis of rumen and ileum tissues (*n* = 3). Representative H&E-stained images of the rumen (**A**–**D**) and ileum (**E**–**H**). (**A**) Group C (Rumen); (**B**) Group L (Rumen); (**C**) Group M (Rumen); (**D**) Group H (Rumen). (**E**) Group C (Ileum); (**F**) Group L (Ileum); (**G**) Group M (Ileum); (**H**) Group H (Ileum). Magnification is indicated by the scale bar in each image.

The histological analysis revealed significant structural changes in the rumen but not in the small intestine (Table 4, Figure 5). Specifically, total ruminal papillae width differed significantly (*p* < 0.05), with group L being greater than group H. No significant differences were found in small-intestinal villus parameters.

### 3.6. Gut Microbiota of Hu Sheep After L. salivarius KS1018 Supplementation

**Figure 6:**
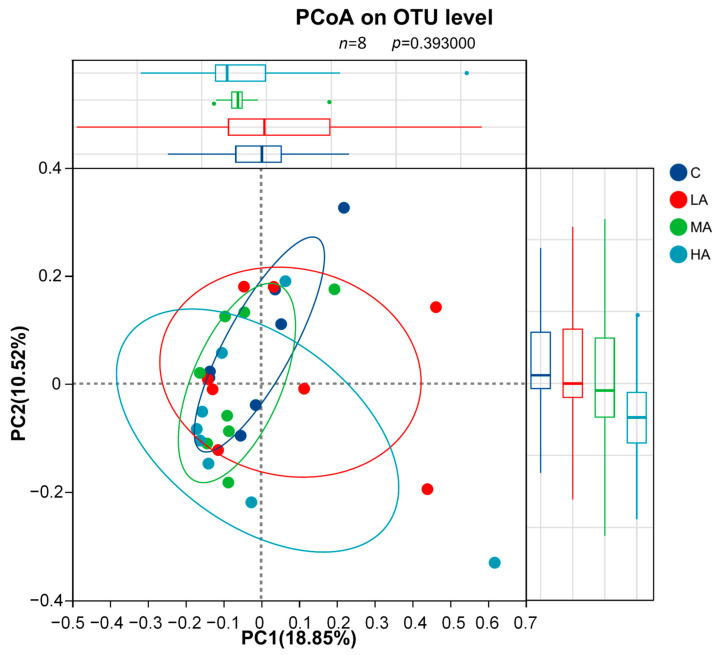
Principal Coordinate Analysis (PCoA) of gut microbiota based on OTU level (*n* = 8). The analysis was performed using Bray–Curtis distance. No significant separation was observed among the four groups (ANOSIM, *p* = 0.393).

A total of 1,414,753 high-quality sequences were obtained with an average of 45,637 sequences per sample (35,114–53,010 sequences). No significant differences were observed in the bacterial α-diversity indices (including the ACE, Chao1, Shannon, and Simpson indices) among four groups (*p* > 0.05). The PCoA at the OTU level with ANOSIM analysis showed no significant differences among groups (R = 0.0081, *p* = 0.393, 999 permutations; Figure 6). Consistently, PERMANOVA revealed no significant separation (F = 1.082, R^2^ = 0.107, *p* = 0.257, 999 permutations).

**Figure 7:**
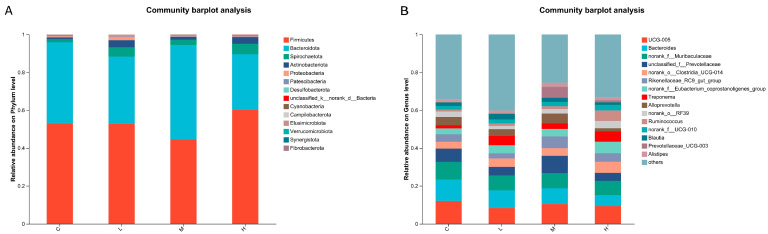
Gut microbiota community composition (*n* = 8). (**A**) Phylum-level microbial composition. (**B**) Genus-level microbial composition.

At the phylum level, the gut microbiota composition of groups supplemented with different amounts of *L. salivarius* KS1018 was characterized (Figure 7A). The community was predominantly composed of Firmicutes and Bacteroidota, with other notable phyla including Spirochaetota, Actinobacteriota, and Patescibacteria. The relative abundance of these bacterial phyla showed no significant differences between three treatment groups and group C. Analysis at the genus level identified the key bacterial taxa within the gut microbiota of Hu sheep (Figure 7B). The relative abundance of these bacterial genera showed no significant differences between three treatment groups and group C.

**Figure 8:**
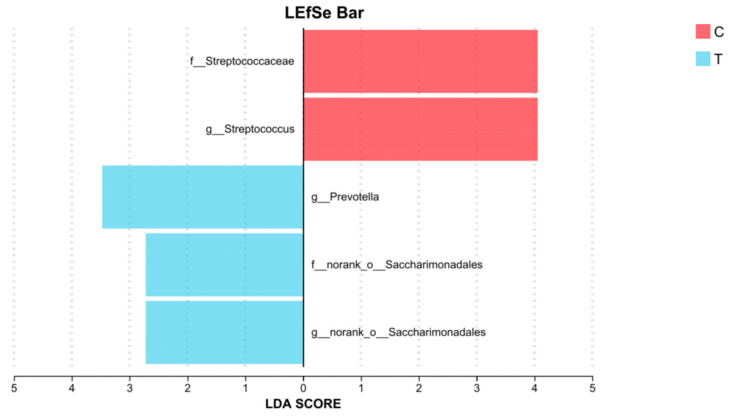
LDA score between group C (*n* = 8) and three treatment groups (group T, *n* = 24) (LDA > 2.5, *p* < 0.05).

LEfSe analysis identified differentially abundant taxa between group C and three treatment groups (T), with an LDA score threshold of 2.5. The results revealed that several taxa were significantly enriched in the control group, including f_*Streptococcaceae*, and *g_Streptococcus*. In contrast, the treatment groups exhibited higher LDA scores for *g_Prevotella*, f_norank_o_*Saccharimonadales*, *g_norank_o_Saccharimonadales* (Figure 8).

### 3.7. Correlation Between Gut Microbiota and Serum Parameters

**Figure 9:**
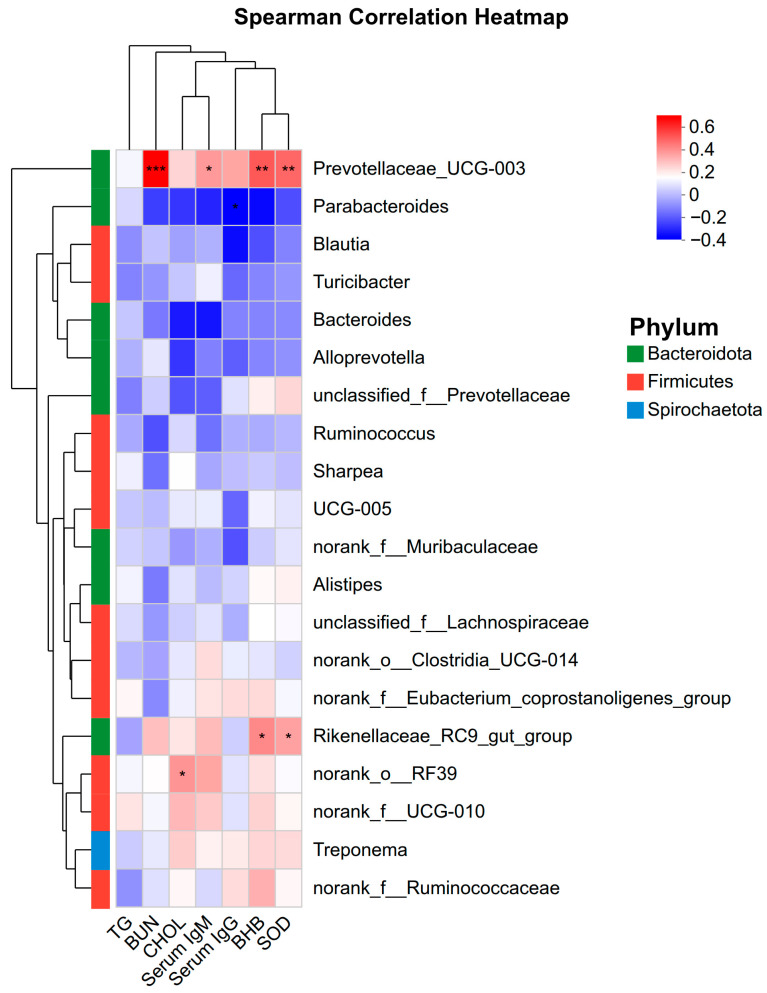
Spearman correlation heatmap between the top 20 abundant bacterial genera and host serum parameters. The color scale represents the correlation coefficient (T), with red indicating positive correlation and blue indicating negative correlation. * *p* < 0.05, ** *p* < 0.01, *** *p* < 0.001. TG: Triglycerides; BUN: Blood Urea Nitrogen; CHOL: Cholesterol; BHB: β-hydroxybutyrate; SOD: Superoxide dismutase.

To explore the functional relationship between the altered gut microbiota and host physiology, Spearman’s correlation analysis was conducted (Figure 9). *Prevotellaceae_UCG-003* showed strong positive correlations with BUN (*p* < 0.001), serum IgM (*p* < 0.05), BHB (*p* < 0.01), and SOD (*p* < 0.01). Additionally, *Rikenellaceae_RC9_gut_group* was positively correlated with BHB (*p* < 0.05) and SOD (*p* < 0.05), while *norank_o_RF39* was positively associated with CHOL (*p* < 0.05). In contrast, *Parabacteroides* exhibited a negative correlation with serum IgG (*p* < 0.05).

## 4. Discussion

The primary objective of this study was to characterize the rumen microbiota of Duolang sheep and evaluate the effects of the derived strain *L. salivarius* KS1018 on the ruminal physiology, blood metabolic profiles, and immune status of Hu sheep. Findings demonstrated the potential of *L. salivarius* KS1018 to enhance antioxidant and immune function in sheep.

This study is the first to provide an overview of the distinct ruminal bacterial profiles of Duolang sheep to gain insight into Duolang sheep’s adaptability to low-quality local roughage. As foregut fermenters, ruminants rely heavily on the microorganisms in the rumen to degrade plant fibers for energy production [25]. *Firmicutes*, *Bacteroidetes* and *Fibrobacteres* are the major ruminal phyla contributing to the breakdown of insoluble carbohydrates in feedstuff [25]. In the present study, the rumen of Duolang sheep contained significantly higher levels of Firmicutes, while Bacteroidetes showed a tendency to be lower compared to Hu sheep. Under the same feeding regimen, an increased ratio of Firmicutes: Bacteroidetes has been related to decreased residual feed intake (RFI) phenotype in sheep [26]. The structural shift in these predominant phyla suggests a potential for higher feed efficiency in Duolang sheep. Several taxa within the phylum Firmicutes, including families UCG-011, Christensenellaceae, and Ruminococcaceae, were significantly enriched in Duolang sheep. They have been reported to be involved in ruminal fiber degradation, biohydrogenation and short-chain fatty acid (SCFA) production as an energy source [27,28]. The higher abundance of these bacterial taxa in Duolang sheep might suggest its enhanced fiber degradation and SCFA production critical for digesting low quality roughage.

Simpson index in Duolang sheep is significantly higher than in Hu sheep, indicating lower level of diversity of ruminal microbiota in Duolang sheep. This is consistent with previous reports about the influence of breed on alpha diversity in ruminants [29,30]. It has been revealed that Cheviot sheep had lower rumen microbial diversity than Lenark sheep did, yet exhibited the higher feed efficiency based on feed conversion ratio (FCR) evaluation [31]. Another report showed that sheep with lower feed efficiency had higher microbial diversity in the liquid rumen fraction [32]. A similar pattern has also been reported in bovine species, where the major hypothesis is that a simpler but more specialized rumen microbiota could better support feed efficiency [33]. In this regard, lower ruminal microbiota diversity of Duolang sheep could be related to higher feed efficiency.

The distinct microbial signatures identified in Duolang sheep rumen suggest a co-evolved microbiota potential associated with high feed efficiency. This unique microbial ecosystem represents a valuable bio-resource for novel, host-adapted probiotic candidates. It was hypothesized that introducing a key beneficial bacterium isolated from this superior environment into Hu sheep could positively influence their gastrointestinal function and overall health. Therefore, *L. salivarius* KS1018 isolated from Duolang sheep was selected as a representative candidate strain. This study investigated the functional effects of *L. salivarius* KS1018 supplementation on the gastrointestinal health and immune status of Hu sheep, specifically examining fecal characteristics, systemic immune and antioxidant status, mucosal SIgA levels, gastrointestinal morphology, and gut microbiota.

Supplementation with *L. salivarius* KS1018 demonstrated positive influence on immune and antioxidant status of Hu sheep. Significantly elevated serum IgG and IgM levels were observed after *L. salivarius* KS1018 supplementation, indicating a potential enhancement of systemic immune function in Hu sheep [34]. SOD enzymes are reliable indicators of oxidative stress and responsible for eliminating excess free radicals, thereby protecting cells from oxidative damage. The significant increase in SOD after *L. salivarius* KS1018 supplementation suggested the increased antioxidant capacity, which is consistent with previous studies on other strains of *L. salivarius* in broilers [35].

Serum biochemical parameters are influenced by various factors, including breed, location and season nutrition and physiological status [36]. In this study, elevations in TG, CHOL, BHB, and BUN were observed. However, these biomarkers generally remained within the established physiological reference ranges for sheep [37], reflecting modest biochemical fluctuations rather than pathological metabolic stress. Direct comparisons of serum biochemistry between Duolang and Hu sheep are currently unavailable. Longer feeding trial may facilitate further investigation of the observed metabolic changes induced by *L. salivarius* KS1018 supplementation.

The most compelling effect of *L. salivarius* KS1018 was its segment-specific regulation of mucosal immunity. Supplementation with the strain significantly increased sIgA levels in the duodenum, a hallmark of enhanced mucosal defense at a critical site for nutrient absorption and pathogen encounter [38]. Even though *L. salivarius* KS1018 did not reveal significant effect on the structure of small intestinal villi or most fecal nutrient parameters, fecal crude fat has increased significantly after supplementation. This suggests that the primary role of *L. salivarius* KS1018 is likely focused on improving gut barrier function and immune health rather than directly enhancing macronutrient digestibility. The change in fecal crude fat may be associated with bile salt hydrolase (BSH) produced by *Lactobacillus* species, an enzyme that influences fat emulsification and absorption, although the specific mechanism requires further metabolomic investigation [39].

To investigate the impact of *L. salivarius* KS1018 on the gut microbiota of Hu sheep, we characterized the fecal microbial community. Dominant phyla such as Firmicutes and Bacteroidota remain relatively stable, indicating their importance in maintaining gut ecological balance [40]. The minimal impact of *L. salivarius* KS1018 supplementation on overall similar microbial diversity suggests that short-term supplementation did not disrupt the intrinsic stability of the gut microbiota. Further long-term studies may help elucidate its effects on microbial diversity over time.

LEfSe analysis revealed an enrichment of taxa from *f_Streptococcaceae*, with a particular emphasis on the *g_Streptococcus*, within group Control. *g_Streptococcus*, often considered opportunistic pathogens, can overgrow and lead to gastrointestinal infections and other health issues [41,42]. Therefore, the use of *L. salivarius* KS1018 may help maintain gut health by reducing the colonization of these potentially pathogenic bacteria. Group Treatment exhibited significantly higher LDA scores for *g_Prevotella*, f_norank_o_Saccharimonadales, and *g_norank_o_Saccharimonadales*. The research on f_norank_o_Saccharimonadales and *g_norank_o_Saccharimonadales* in the gut is limited, while *g_Prevotella* is known to ferment plant fibers to produce SCFA which can provide energy [43] and may participate in immune processes [44]. This evidence further suggests that *L. salivarius* KS1018 may influence gut immune activities and the availability of microbial-derived nutrients in the gut.

To further explore the mechanism, correlation analyses on the fecal microbiota were integrated, which highlighted *Prevotellaceae_UCG-003* as a taxon positively correlating with BUN, BHB, IgM, and SOD. Members of the *Prevotellaceae* family are functionally recognized for their role in hemicellulose degradation and protein breakdown [45,46,47]. Consequently, the positive correlation between *Prevotellaceae_UCG-003* and these serum metabolites suggests that the altered microbiota may be involved in modulating nitrogen and energy status. Future studies utilizing metabolic cages and tracer techniques are necessary to directly verify whether the association will improve whole-tract nutrient digestibility.

Limitations of the present study have to be acknowledged. First, the sample sizes for histological analysis (*n* = 3) as well as serum profiles and gut microbiota analyses (*n* = 8) were limited, reducing the power to analyze treatment effects despite the use of strict inclusion criteria to control inter-animal variability. Second, baseline (Day 0) measurements were not collected, restricting intra-individual comparisons; nevertheless, the randomized parallel-group design enabled evaluation of endpoint differences attributable to treatment, although we acknowledge that the group-housing setting implies that exact individual dosage uniformity could not be strictly guaranteed. Third, the absence of complementary approaches such as metabolomics or transcriptomics restricts interpretation of the functional mechanisms underlying the effects of *L. salivarius* KS1018. Future studies integrating multi-omics analyses are needed to provide more insight.

## 5. Conclusions

The study characterized the distinct rumen microbiota of the highly adapted Duolang sheep, which provided the basis for validating the targeted effects of *L. salivarius* KS1018. In Hu sheep, supplementation with this strain improved immune status and elevated antioxidant capacity while preserving overall microbial community stability. These findings highlight the significant potential of using host-adapted probiotics to influence host physiology including gastrointestinal function, immunity and overall health. Further research into the underlying mechanisms of probiotic action will promote more effective utilization of the potential probiotic strains.

## Figures and Tables

**Table 1:** Comparison of relative abundance of dominant bacterial taxa in ruminal fluid from Hu sheep and Duolang sheep.

Taxonomy Level	Taxa	Relative Abundance %		Adjusted *p*-Value
		Hu Sheep	Duolang Sheep	
Phylum	*Bacteroidetes*	48.76 ± 10.44	39.62 ± 6.36	0.05372 ^†^
	*Firmicutes*	44.45 ± 10.84	56.45 ± 6.52	0.02707 *
	*Proteobacteria*	1.82 ± 1.73	0.41 ± 0.19	0.04011 *
	*Patescibacteria*	1.13 ± 0.45	0.64 ± 0.31	0.02746 *
Family	*Prevotellaceae*	24.39 ± 10.50	15.64 ± 5.08	0.05804 ^†^
	*Oscillospiraceae*	6.82 ± 2.81	9.06 ± 1.33	0.06423 ^†^
	*UCG-011*	5.29 ± 1.67	9.23 ± 3.35	0.01889 *
	*Christensenellaceae*	4.43 ± 1.51	6.07 ± 1.20	0.04744 *
	*F082*	4.31 ± 0.97	5.92 ± 1.76	0.04744 *
	*Ruminococcaceae*	3.76 ± 0.89	5.86 ± 2.48	0.04744 *
Genus	*Prevotella*	18.66 ± 8.16	12.05 ± 3.71	0.07889 ^†^
	*unclassified UCG-011*	5.29 ± 1.67	9.23 ± 3.35	0.02316 *
	*Christensenellaceae R7 group*	4.39 ± 1.50	6.02 ± 1.19	0.05362 ^†^
	*unclassified F082*	4.31 ± 0.97	5.92 ± 1.76	0.06523 ^†^
	*Ruminococcus*	2.30 ± 0.52	3.58 ± 1.68	0.07889 ^†^

Data are presented as Mean ± SD (*n* = 12). *p*-values were adjusted for multiple comparisons using the Benjamini–Hochberg (FDR) method. * *p* < 0.05, ^†^ 0.05 < *p* < 0.10 indicates a statistical tendency.

**Table 2:** Effects of *L. salivarius* KS1018 supplementation on fecal characteristics in Hu sheep (*n* = 8).

Fecal Characteristics	C	L	M	H	*p*-Value
Moisture, %	31.83 ± 3.62	31.07 ± 5.21	31.04 ± 5.04	32.39 ± 8.48	0.9620
Ash, %	17.73 ± 1.24	18.76 ± 3.36	21.38 ± 4.11	19.85 ± 4.20	0.2337
Crude protein, %	2.98 ± 0.21	3.15 ± 0.30	3.23 ± 0.24	2.90 ± 0.32	0.0870
Ether extract, %	3.08 ± 0.75 ^ab^	2.82 ± 1.29 ^b^	4.43 ± 1.13 ^a^	2.63 ± 1.02 ^b^	0.0099 **
Crude fiber, %	14.95 ± 1.60	13.00 ± 2.45	15.48 ± 2.23	12.98 ± 3.57	0.1380
NDF, %	45.68 ± 3.72	43.25 ± 4.09	44.77 ± 5.09	49.38 ± 4.76	0.0642
ADF, %	32.94 ± 4.88	28.24 ± 4.08	31.05 ± 5.36	35.20 ± 7.56	0.1147
Digestible energy, kJ/100 g	149.76 ± 17.63	172.27 ± 68.63	184.63 ± 66.96	194.63 ± 76.07	0.5602
Fecal score	2.57 ± 0.53	2.25 ± 1.16	2.50 ± 0.53	1.75 ± 1.16	0.2750

Data are presented as Mean ± SD. ** *p* < 0.01. ^a,b^ Means within a row with different superscripts differ (*p* < 0.05).

**Table 3:** Effects of *L. salivarius* KS1018 supplementation on sIgA levels of the rumen, small intestine in Hu sheep (*n* = 3).

sIgA Levels	C	L	M	H	*p*-Value
Rumen, μg/mL	1.96 ± 0.23 ^a^	1.52 ± 0.08 ^b^	1.59 ± 0.04 ^b^	1.50 ± 0.08 ^b^	0.0093 **
Duodenum, μg/mL	1.42 ± 0.52 ^a^	2.20 ± 0.19 ^a^	2.97 ± 0.42 ^ab^	3.18 ± 0.35 ^b^	0.0021 **
Jejunum, μg/mL	3.02 ± 0.79	3.03 ± 0.75	3.49 ± 0.49	3.84 ± 0.66	0.4555
Ileum, μg/mL	2.84 ± 1.00	3.17 ± 1.04	3.53 ± 0.67	3.29 ± 0.27	0.8621

Data are presented as Mean ± SD. ** *p* < 0.01. ^a,b^ Means within a row with different superscripts differ (*p* < 0.05).

**Table 4:** Effects of *L. salivarius* KS1018 supplementation on histological evaluation of the rumen, small intestine in Hu sheep (*n* = 3).

Histological Evaluations	C	L	M	H	*p*-Value
Rumen					
Stratum corneum thickness (μm)	11.03 ± 2.20	10.54 ± 2.69	11.01 ± 2.55	10.70 ± 1.42	0.6858
Connective tissue width (μm)	159.99 ± 110.85	152.68 ± 55.43	151.74 ± 42.85	148.90 ± 47.57	0.7906
Non-keratinized epithelium (μm)	127.11 ± 30.07	140.26 ± 53.69	118.29 ± 36.87	112.42 ± 43.30	0.3727
Total papilla width (μm)	493.25 ± 67.28 ^b^	545.55 ± 107.44 ^a^	470.69 ± 83.81 ^ab^	460.23 ± 73.50 ^b^	0.0363 *
Ileum					
Villus height (μm)	361.66 ± 96.88	303.3 ± 76.23	389.7 ± 103.29	360.6 ± 82.14	0.1779
Crypt depth (μm)	214.89 ± 65.61	201.31 ± 42.52	244.53 ± 39.42	236.89 ± 70.75	0.2619
Villus/crypt ratios	1.72 ± 0.30	1.53 ± 0.34	1.59 ± 0.28	1.74 ± 0.40	0.4543

Data are presented as Mean ± SD. * *p* < 0.05. ^a,b^ Means within a row with different superscripts differ (*p* < 0.05).

## Data Availability

The original data presented in the study are openly available in FigShare at 10.6084/m9.figshare.24891966.

## Supplementary Materials

The following supporting information can be downloaded at: https://www.mdpi.com/article/10.3390/vetsci12121177/s1, Table S1: Chemical analysis of TMR ingredients.

## Abbreviations

The following abbreviations are used in this manuscript:

ACE	Abundance-based coverage estimator
ADF	Acid detergent fiber
ANOSIM	Analysis of similarities
ANOVA	Analysis of variance
BHB	β-hydroxybutyrate
BSFS	Bristol stool form scale
BSH	Bile salt hydrolase
BUN	Blood urea nitrogen
CF	Crude fiber
CFU	Colony-forming unit
CHOL	Total cholesterol
CP	Crude protein
DE	Digestible energy
DM	Dry matter
EE	Ether extract
ELISA	Enzyme-linked immunosorbent assay
FCR	Feed conversion ratio
FDR	False discovery rate
H&E	Hematoxylin and eosin
IgG	Immunoglobulin G
IgM	Immunoglobulin M
*L. salivarius*	*Ligilactobacillus salivarius*
LDA	Linear discriminant analysis
LEfSe	Linear discriminant analysis effect size
NDF	Neutral detergent fiber
OTU	Operational taxonomic unit
PCoA	Principal coordinate analysis
PCR	Polymerase chain reaction
RDP	Ribosomal database project
RFI	Residual feed intake
SCFA	Short-chain fatty acid
sIgA	Secretory immunoglobulin A
SOD	Superoxide dismutase
TG	Triglyceride
TMR	Total mixed ration
