# Supplementary material for: Host-Adapted Ruminal Microbiota Investigation and Functional Validation of Duolang Sheep-Derived Ligilactobacillus salivarius KS1018
Source: Vet Sci. 2025 Dec 10;12(12):1177. doi: 10.3390/vetsci12121177 (PMC12737677; doi:10.3390/vetsci12121177)
Supplement: Supplementary file 1 [file vetsci-12-01177-s001.zip › vetsci-3984088-supplementary.pdf]

Supplementary Table S1. Chemical analysis of TMR ingredients

No.	Ingredient	Unit	Result	Testing standard
1	Crude Protein	%	5.59	GB/T 6432-2018
2	Calcium	%	0.323	GB/T 6436-2018
3	Total Phosphorus	%	0.18	GB/T 6437-2018
4	Vitamin A	IU/kg	1065.1	GB/T 17817-2010
5	Vitamin D3	IU/kg	1763.9	GB/T 17818-2010
6	Vitamin E	IU/kg	15.6	GB/T 17812-2008
7	Nicotinic acid	mg/kg	164.6	GB/T 17813-2018 (3)
8	Iron	mg/kg	8.27	GB/T 13885-2017
9	Manganese	mg/kg	12.6	GB/T 13885-2017
10	Copper	mg/kg	7.17	GB/T 13885-2017
11	Zinc	mg/kg	11.8	GB/T 13885-2017
12	Selenium	mg/kg	0.283	GB/T 13883-2008 (Method 1)
13	NDF (neutral detergent fiber)	%	62.0	GB/T 20806-2006
14	ADF (acid detergent fiber)	%	34.5	NY/T 1459-2022
15	Cobalt	mg/kg	1.50	GB/T 13884-2018
16	Ether extract	g/kg	6	GB/T 6433-2006
17	Non-Fiber Carbohydrate	%	11.3	GB/T 6434-2006
18	Dry Matter	%	64.9	GB 5009.3-2016
19	Iodine	mg/kg	1.04	GB/T 13882-2010
